# RMP-YOLO: Robust Multi-Scale Pedestrian Detection for Dense Scenarios

**DOI:** 10.3390/s26092621

**Published:** 2026-04-23

**Authors:** Chenyang Gui, Zhangyu Fan, Taibin Duan, Junhao Wen

**Affiliations:** 1 School of Big Data and Software Engineering, Chongqing University, Chongqing 400044, China; 2 School of Aerospace Engineering, Xi’an Jiaotong University, Xi’an 710049, China; 3 School of Mechanical Engineering and Automation, Fuzhou University, Fuzhou 350108, China; 4 School of Mathematical and Physical Sciences, Chongqing University of Science and Technology, Chongqing 401331, China

**Keywords:** high precision, MobileViTv3, PIoUv2, RFAConv

## Abstract

With the rapid advancement of autonomous driving in modern society, dense pedestrian detection technology has encountered performance bottlenecks. To address this, we propose a robust and lightweight pedestrian detection algorithm, RMP-YOLO, designed to efficiently detect small, occluded, and low-light objects. Firstly, RFAConv is utilized as the core component of the backbone network, combining standard convolution with attention mechanisms and using group convolution to extract features from the spatial receptive field. Secondly, MobileViTv3 is introduced into the backbone to combine CNNs with Transformers. The model is further enhanced by adjusting feature fusion, introducing residual connections, and optimizing local representation with deep convolutional layers. Finally, the PIoUv2 loss function is employed for bounding-box regression, significantly reducing detection errors for small-scale pedestrians in crowded environments. Experimental results demonstrate that RMP-YOLO improves mAP@0.5 by 1.3% on a custom dataset and 0.91% on the WiderPerson dataset. Crucially, it maintains high efficiency with only 3.71 million parameters and 6.29 GFLOPs, meeting the deployment requirements for low computational power and high precision.

## 1. Introduction

Currently, global intelligent connected vehicle technology is rapidly evolving, and pedestrian detection and recognition are key components of vehicle active safety systems. It has become a focal research area in both academia and industry. In traffic accidents, casualties resulting from delayed or incorrect pedestrian detection and recognition are recurring. According to statistics from the World Health Organization, road traffic deaths were estimated at approximately 1.19 million in 2021, with pedestrians accounting for 21% of the deaths [1]. This is a relatively high proportion, particularly in pedestrian-dense areas, which often carry greater safety risks. Moreover, drivers’ reaction times and distracted attention typically fail to respond effectively to sudden situations. Therefore, improving the accuracy, computational efficiency, robustness, and generalization capability of pedestrian detection and recognition to reduce traffic accidents holds significant social importance.

Autonomous vehicles are seen as a crucial technological solution to traffic accidents, and features such as collision warning have already been proven to reduce traffic accident rates by up to 33% [2]. On the technical side, collision warning functions are closely tied to pedestrian detection and recognition, yet this is a highly complex computer vision task with numerous challenges. For instance, challenges include varying lighting levels, occlusion, and the small size of pedestrian targets. Traditional methods are no longer suitable for these complex scenarios, as techniques such as HOG and SVM are increasingly unable to meet the requirements of modern pedestrian detection. However, new methods and solutions for effective and precise pedestrian identification and recognition have emerged with the development of deep learning technologies, particularly convolutional neural networks and object detection algorithms such as YOLO [3], SSD [4], and Faster R-CNN [5]. For instance, Convolutional Neural Networks (CNNs) can self-learn target features and exhibit better robustness and generalization [6].

To address the above challenges, this study proposes RMP-YOLO, a lightweight, unified framework for dense pedestrian detection that accounts for the joint influence of small targets, severe occlusion, and low-light conditions. The proposed method aims to improve detection robustness and generalization while maintaining low computational complexity within a compact YOLOv8-based architecture. The detailed network design and module implementation are presented in Section 3.

The main contributions of this work are summarized as follows:

- A lightweight unified framework, termed RMP-YOLO, is proposed for dense pedestrian detection under the joint influence of small targets, severe occlusion, and low-light conditions.
- A compact YOLOv8-based architecture is constructed by integrating RFAConv [7], MobileViTv3 [8], and PIoUv2 [9] to enhance feature extraction, context modeling, and bounding-box regression for dense pedestrian scenes.
- Extensive experiments, including visualization analysis, ablation studies, comparative evaluations, and cross-dataset validation, verify that the proposed method improves dense pedestrian detection performance while maintaining low computational complexity.

## 2. Related Work

Pedestrian detection has long been a fundamental task in intelligent transportation and crowded-scene perception. In dense pedestrian scenarios, many studies have focused on alleviating occlusion and feature confusion caused by severe overlap among adjacent instances. For example, Zhang et al. [10] proposed Occlusion-aware R-CNN to suppress interference among neighboring pedestrians, and Ge et al. [11] improved dense pedestrian detection by introducing a loss-aware label assignment strategy. Zhang et al. [12] further enhanced robustness under heavy occlusion through a variational learning framework. Based on the YOLO family, Wu [13] investigated a YOLOv5-based dense pedestrian detection method, while Li et al. [14] developed a lightweight YOLOv7-based detector for obscured pedestrians. For dense scenes, Wang et al. [15] improved YOLOv8 by introducing MobileViT, EMA, and Repulsion Loss, whereas Jiang et al. [16] enhanced YOLOv8 with SimAm attention. These methods demonstrate that tailored architectural enhancement can effectively improve feature discrimination and localization in crowded scenes. However, most of them mainly target one dominant challenge, such as occlusion reasoning, dense overlap suppression, or small-object enhancement, and their improvements are often confined to specific conditions rather than a unified dense pedestrian perception framework.

Low-light environments further increase the difficulty of pedestrian detection because insufficient illumination weakens object contrast, destroys texture details, and aggravates background interference. To address this problem, Mao [17] improved dense crowd pedestrian detection in low-light scenes using an enhanced YOLO-based framework, while Cui et al. [18] combined bright-channel prior attention and multispectral cues to strengthen perception under poor illumination. Yi et al. [19] incorporated an improved YOLOX model with domain transfer strategies for nighttime pedestrian and vehicle detection, and Lai et al. [20] optimized Mask R-CNN for pedestrian detection in low-light road environments. In related vision tasks, Lei et al. [21] reduced background-induced domain shift in person re-identification, and Liu et al. [22] improved subterranean pedestrian detection through domain-adaptive transfer and confidence guidance. These studies confirm that illumination enhancement, domain adaptation, and feature refinement are all effective for improving perception quality in weak-light scenes. Nevertheless, many existing approaches are still highly scenario-dependent, and their robustness tends to decline when low illumination is coupled with small pedestrians, dense overlap, and severe occlusion, which are common in real-world road environments.

In addition to occlusion and illumination, small-target perception and lightweight deployment are also crucial for practical intelligent driving systems. Fang and Pang [23], Li et al. [24], and Ye and Wang [25] improved dense-scene or small-object detection by introducing adaptive feature fusion, dynamic convolution, and Transformer-assisted representation, respectively. From the perspective of intelligent driving, robust perception should also support downstream autonomous driving decision-making [26]. Therefore, a series of lightweight and attention-enhanced methods have been explored, including improved YOLOv5-based dense pedestrian detectors [27], RepVGG-based efficient structures [28], and ECA-based channel attention [29]. Wei et al. [30] proposed YOLO-Person for pedestrian detection in road areas, while Fayyad et al. [31] reviewed deep-learning-based sensor fusion for autonomous vehicle perception and localization. In addition, CBAM [32] verified the effectiveness of lightweight attention mechanisms for enhancing salient feature learning. Overall, existing studies have provided valuable foundations for pedestrian detection in terms of occlusion handling, illumination adaptation, small-target enhancement, and lightweight design. Their main advantage lies in improving one or several stages of the detection pipeline through carefully designed modules. However, methods with stronger robustness often introduce additional complexity, depend on auxiliary priors or extra modalities, or fail to balance local detail extraction, global feature interaction, localization quality, and deployment efficiency simultaneously. Therefore, there remains a need for a compact and unified framework that can jointly address dense overlap, small targets, low-light interference, and lightweight deployment requirements, which is the main motivation for the proposed RMP-YOLO.

In recent years, synthetic-data generation and simulated environments have emerged as a promising direction in computer vision, especially when real-world data are difficult to collect or insufficiently diverse. Staniszewski et al. [33] showed that properly designed synthetic data can effectively complement real-world datasets and improve detection robustness, particularly when data diversity and photorealism are well controlled. For dense pedestrian detection, such a strategy is potentially useful for generating controllable training samples with varying crowd densities, occlusion patterns, lighting conditions, and weather conditions. However, the effectiveness of synthetic data still depends on reducing the domain gap between simulated and real scenes.

## 3. Method

### 3.1. Baseline Architecture

The YOLO series of object detectors has undergone continuous evolution, consistently aiming to balance competitive detection accuracy with computational efficiency. The seminal YOLOv1 pioneered reformulating object detection as a single-stage regression problem. Subsequent iterations, including YOLOv2 [34] and YOLOv3 [35], significantly enhanced detection performance across varying object scales by incorporating anchor-based mechanisms and multi-scale feature fusion. YOLOv4 [36] and YOLOv5 [37] further consolidated the framework’s industrial dominance by integrating advanced architectural components, such as CSPNet and PANet, and robust data augmentation strategies, such as Mosaic. More recent versions have increasingly prioritized model flexibility and computational efficiency across diverse hardware constraints.

**Figure 1:**
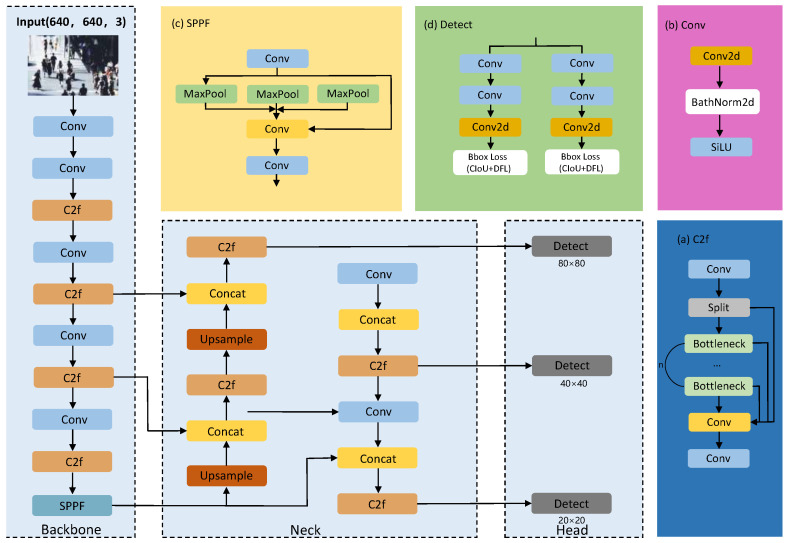
YOLOv8 framework structure.

In this study, YOLOv8 is selected as the baseline architecture. Its overall framework, shown in Figure 1, comprises three primary components: the Backbone, Neck, and Head. A distinctive improvement over its predecessors is the adoption of an anchor-free detection strategy. This approach alleviates reliance on predefined anchor boxes, thereby enhancing the model’s generalization capabilities. Within the Backbone and Neck, YOLOv8 introduces the C2f module to supersede the C3 module used in YOLOv5. Synthesizing the design principles of CSP and ELAN, the C2f module utilizes enriched skip connections to facilitate superior gradient flow. This design effectively bolsters feature extraction capabilities while maintaining a lightweight computational footprint. Furthermore, YOLOv8 employs a decoupled head structure to process classification and regression tasks independently and incorporates a Task Aligned Assignor to optimize positive and negative sample assignment. Building upon the core framework of YOLOv8, this paper introduces improvements to propose the RMP-YOLO algorithm.

**Figure 2:**
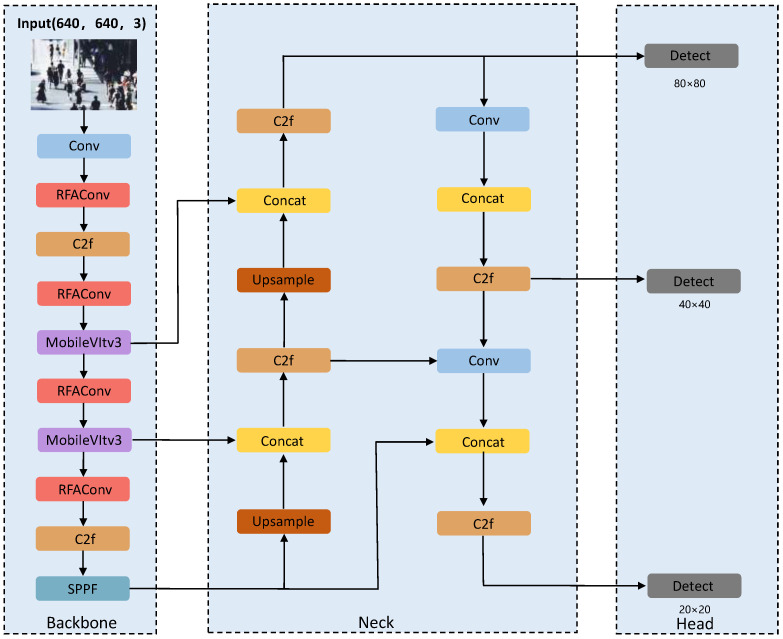
RMP-YOLO framework structure.

Although YOLOv8 achieves strong overall detection performance, it still suffers from insufficient retention of small-target features, limited robustness under severe occlusion and low-light conditions, and suboptimal localization quality in dense pedestrian scenes. To address these issues, this paper improves the YOLOv8 baseline by incorporating receptive-field-aware feature extraction, lightweight global–local representation learning, and bounding-box regression optimization, thereby proposing the RMP-YOLO algorithm. The overall framework of RMP-YOLO is shown in Figure 2.

### 3.2. RFAConv

By prioritizing spatial receptive field features, the receptive field feature-weighted convolution assigns relative importance to each feature within the receptive field [7]. Specifically, it generates Receptive-Field Attention Maps to weight features within the sliding region, focusing on the receptive field’s spatial attributes. The spatial features of the receptive field are represented by feature maps extracted by RFA. After shape adaptation, there are no overlapping issues, and the learned attention map integrates the feature data from each sliding window within the receptive field. The attention map for each receptive-field sliding window is computed independently. The receptive-field spatial feature is a special feature map that captures the convolutional kernel’s response at different locations. It represents the original feature map after the targeted transformation, generated using a non-overlapping sliding-window method. This section explains how a 3×3 window in the spatial features represents the receptive field sliding. The receptive field features are adapted to the convolution kernel structure and automatically adjusted according to the kernel size. The term “spatial features” refers to the original feature mapping. Using the initial spatial features as a foundation, a non-overlapping sliding-window transformation is applied using a 3×3 convolution kernel. After transformation, the receptive field feature space is generated, and the resulting feature map has the spatial dimensions of C × 3H × 3W. A single 3×3 window represents the sliding process of a receptive field.

After adopting the RFA structure, this approach extracts receptive-field spatial characteristics using group convolutions, employing a lightweight strategy to promote information exchange within the receptive field, thereby optimizing network performance with only a limited additional computational cost and parameter overhead. Global information from receptive field features is integrated via AvgPool, and l×l group convolutions are employed to enable feature interaction. The softmax function normalizes the weight ratios for each feature within the receptive field. The formula of RFA is defined as follows [7]:

(1) $$F=\mathrm{Softmax}\left(g^{1\times 1}\left(\mathrm{AvgPool}\left(X\right)\right)\right)\times \mathrm{ReLU}\left(\mathrm{Norm}(g^{k\times k}\left(X\right))\right)=A_{\mathrm{rf}}\times F_{\mathrm{rf}}$$

The symbol $g^{1\times 1}$ represents a group convolution with a kernel size of 1×1, designed to facilitate information interaction across the receptive field. In the proposed architecture, RFAConv replaces standard convolutions; therefore, the kernel size is set to k=3. The function Norm denotes the Batch Normalization operation, which helps stabilize training. Let *X* be the input feature map. The final output *F* is obtained by performing an element-wise multiplication between the transformed receptive-field features, denoted as $F_{\mathrm{rf}}$, and the attention map $A_{\mathrm{rf}}$, which is derived from the Softmax branch.

The problem of shared kernel parameters in earlier models is successfully addressed by RFAConv, which incorporates spatial receptive field data into the convolutional process. This allows the network to more precisely capture positional differences and fine-grained image details. Such an approach minimizes increases in computational cost and parameter count, significantly enhancing network performance. The RFAConv module employs group convolutions that, while mapping the original feature map to new feature maps, also reduce computational overhead. Average pooling aggregates global data across all receptive fields, thereby further increasing efficiency. This reduces redundant information in feature processing, thereby decreasing computational complexity.

### 3.3. MobileViTv3

Due to the powerful capabilities of CNNs and EfficientNet in classification, detection, and visual segmentation tasks, they have been widely applied across various fields. However, when deploying CNNs on resource-constrained devices, MobileViTv3 demonstrates its outstanding value. As a hybrid architecture designed for lightweight vision tasks, MobileViTv3 effectively addresses the limitations of traditional lightweight models by leveraging collaborative mechanisms between CNNs and Transformers. By efficiently integrating local, global, and input features, MobileViTv3 significantly enhances performance while maintaining low parameter counts and a low number of floating-point operations. It leverages Transformers’ global feature modeling capabilities alongside convolutional neural networks’ local feature extraction abilities. Integrating linear-complexity Transformers and optimized feature fusion enables outstanding performance. Unlike standard Multi-Head Self-Attention, which exhibits quadratic complexity $O(N^{2})$ due to pairwise matrix computations, the Separable Self-Attention reduces the complexity to linear O(N) with respect to the number of tokens.

Specifically, it replaces the matrix multiplication with element-wise operations by generating a latent context vector. This vector encodes global information and is efficiently broadcast to interact with input tokens, allowing the model to capture long-range dependencies with significantly lower computational cost. This lightweight design makes it attractive for complexity-constrained vision applications.

The MobileViTv3 module comprises three core constituents: a local-representation branch, a global-representation branch, and a fusion stage. Its input feature map is shaped as $C_{in}\;\;\;\times \;\;\;W\;\;\;\times \;\;\;H$, with $C_{in}$ indicating the number of channels and *W*, *H* corresponding to spatial width and height, respectively. First, the local module extracts fine-grained features from the input using both standard and depthwise separable convolutions. Here, the depthwise path independently applies convolution to each input channel. Next, the global module captures contextual information from the entire feature map through a Transformer sub-module—performing self-attention—and a convolution sub-module that reshapes the feature dimensions in preparation for fusion. The fusion module then merges the outputs of both the local and global modules, enriching spatial and positional cues before applying a convolutional operation to further refine the concatenated features. The final feature map $C_{out}\times H\times W$ is generated by combining the fused output with the original input map.

### 3.4. PIoUv2

In object detection, precise localization hinges on bounding-box regression, whose efficacy is primarily dictated by the choice of loss function. Current IoU evaluation methods employ an unreasonable penalty mechanism, leading to excessive growth in anchor box sizes during regression and slowing overall convergence. To mitigate this concern, the study adopts Power-IoUv2 as the bounding-box regression loss. It is an error function for bounding-box regression that uses efficient path regression and non-monotonic focusing mechanisms. The method combines a gradient adjustment scheme driven by anchor box quality with a target size-sensitive penalty factor. This effectively addresses the sluggish convergence speed and anchor box expansion problems associated with conventional IoU losses.

To address the limitations of traditional distance metrics, PIoU introduces an adaptive penalty factor *P* that scales based on the target size. We define the predicted box as B=[x,y,w,h] and the ground truth box as $B_{gt}=\left[x_{gt},y_{gt},w_{gt},h_{gt}\right]$, where (x,y) denote the center coordinates and (w,h) denote the width and height. The absolute distances between the corresponding edges of the predicted box and the ground truth box are calculated as follows:

(2) $$\begin{array}{cccc} d_{w1} & =\left|x-x_{gt}-\frac{w-w_{gt}}{2}\right|\; & d_{h1} & =\left|y-y_{gt}-\frac{h-h_{gt}}{2}\right|\\ d_{w2} & =\left|x-x_{gt}+\frac{w-w_{gt}}{2}\right|\; & d_{h2} & =\left|y-y_{gt}+\frac{h-h_{gt}}{2}\right| \end{array}$$

$dw_{1},dw_{2}$ represent the deviations of the left and right edges, while $dh_{1},dh_{2}$ represent the deviations of the top and bottom edges. The penalty factor *P* is then formulated by normalizing these deviations by the target box’s dimensions [9]:

(3) $$P=\frac{1}{4}\left(\frac{d_{w1}}{w_{gt}}+\frac{d_{w2}}{w_{gt}}+\frac{d_{h1}}{h_{gt}}+\frac{d_{h2}}{h_{gt}}\right)$$

This normalization acts as a size-adaptive scaling mechanism. For smaller targets, a slight positional deviation results in a larger *P*, effectively preventing the “small object box collapse” issue. When there is no overlap, *P* remains strictly positive. To prioritize medium-quality anchors and further refine the gradient allocation during box regression, we add a non-monotonic attention layer on top of PIoU. The attention function u(x) and the quality term *q* are defined as [9]:

(4) $$u\left(x\right)=3x\cdot e^{-x^{2}},\;q=e^{-P},\;q\in \left(0,1\right]$$

Consequently, the final PIoUv2 loss function is expressed as [9]:

(5) $$L_{\mathrm{PIoU\_v2}}=u\left(\lambda q\right)\cdot L_{\mathrm{PIoU}}=3\cdot \left(\lambda q\right)\cdot e^{-{\left(\lambda q\right)}^{2}}\cdot L_{\mathrm{PIoU}}$$

Unlike monotonic weighting strategies, the non-monotonic attention in PIoUv2 does not continuously increase or decrease with anchor quality. Instead, it assigns the highest regression weight to medium-quality anchors, which usually have the greatest optimization potential. Very low-quality anchors are often associated with ambiguous or noisy localization in dense and occluded scenes, and over-emphasizing them may destabilize training. In contrast, very high-quality anchors are already close to the target box and contribute limited further gain. Therefore, the non-monotonic design helps the model focus on the most informative and correctable samples, improving both convergence efficiency and localization quality.

### 3.5. Synergistic Integration of Modules

The interaction among RFAConv, MobileViTv3, and PIoUv2 can be interpreted as a complementary optimization process within the YOLOv8 framework. To describe this relationship more clearly, the detector can be abstractly represented as

(6) $$F_{R}=R_{\theta_{R}}\left(X\right),\;F_{M}=M_{\theta_{M}}\left(F_{R}\right),\;\hat{Y}=H_{\theta_{H}}\left(N\left(F_{M}\right)\right)$$

and the corresponding training objective is

(7) $$L=L_{cls}+\alpha L_{obj}+\beta L_{PIoUv2}$$

In this process, RFAConv is responsible for improving the reliability of local feature extraction. By introducing receptive-field-aware adaptive weighting into standard convolution, it allows different positions within the same receptive field to contribute differently to the final response:

(8) $$y_{r}^{RFA}=\sum_{j=1}^{k^{2}}A_{r,j}K_{j}x_{r,j}$$

This makes local representations more expressive and helps retain weak pedestrian cues in low-illumination and small-object conditions.

On this basis, MobileViTv3 further enriches the features through local–global fusion:

(9) $$F_{M}=F_{R}+\phi \left(\left[F_{l},F_{g}\right]\right)$$

where $F_{l}$ and $F_{g}$ denote local and global representations, respectively. This design allows the network to preserve local details while introducing broader contextual information, which is especially useful for dense pedestrian scenes with frequent occlusion and ambiguity.

PIoUv2 complements feature extraction from the optimization side. Its geometry-aware regression is more sensitive to the localization quality of small pedestrians because the penalty is normalized by the target size. In addition, its non-monotonic focusing mechanism emphasizes medium-quality anchor boxes, which are common in low-light and occluded pedestrian scenes.

More importantly, the supervision introduced by PIoUv2 is propagated backward through the entire detection pipeline. For the RFAConv parameters, the corresponding gradient can be expressed as

(10) $$\nabla_{\theta_{R}}L=\frac{\partial L}{\partial \hat{Y}}\frac{\partial \hat{Y}}{\partial F_{M}}\frac{\partial F_{M}}{\partial F_{R}}\frac{\partial F_{R}}{\partial \theta_{R}}$$

This indicates that localization-oriented supervision also influences upstream feature learning, encouraging RFAConv and MobileViTv3 to produce representations that are both semantically discriminative and localization-sensitive. Therefore, the three modules work together as a collaborative pipeline of local detail enhancement, contextual semantic refinement, and geometry-aware optimization.

## 4. Results

### 4.1. Dataset

To comprehensively evaluate the performance, robustness, and generalization ability of RMP-YOLO, this study uses one custom-made dataset and five public datasets for training and/or evaluation, as summarized in Table 1, which summarizes the scale of each dataset and its usage in this study. In particular, the custom-made Person dataset is used as the primary dataset for training and in-domain evaluation, while the public datasets serve as standardized benchmarks for supplementary evaluation and cross-dataset generalization analysis.

**Figure 3:**
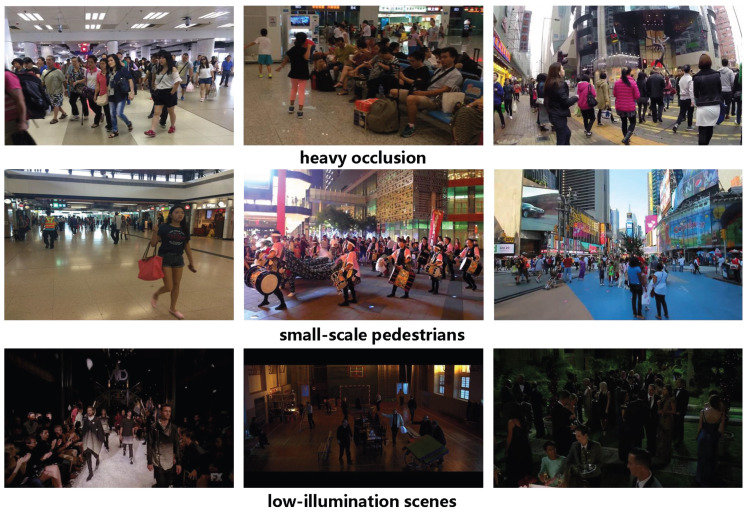
Representative samples from the custom-made Person dataset, illustrating three challenging scenarios: heavy occlusion, small-scale pedestrians, and low illumination.

The custom-made dataset, named Person, contains 18,184 images collected from two sources. The first part consists of real-world photographs captured in densely populated environments, such as shopping malls, subway stations, and pedestrian streets. The second part consists of Internet images deliberately selected to include challenging conditions, especially severe occlusion, small-scale pedestrians, and low-illumination scenes. All pedestrian instances were manually annotated using the open-source tool LabelImg to ensure accurate bounding-box labels. The purpose of constructing the Person dataset is to better reflect the practical detection difficulties emphasized in this work and to provide a dedicated benchmark for evaluating the model under complex pedestrian scenarios. Representative examples from the Person dataset are shown in Figure 3, which illustrate the three major challenging conditions emphasized in this work: heavy occlusion, small targets, and low illumination.

In addition to the Person dataset, three public pedestrian datasets, namely WiderPerson, CityPersons, and CrowdHuman, are adopted to evaluate the model under different crowd characteristics. WiderPerson contains diverse outdoor pedestrian scenes with large variations in scale, pose, and background, making it suitable for testing the detector’s adaptability in unconstrained real-world environments. CityPersons focuses on urban street scenes and is widely used to evaluate pedestrian detection in traffic and roadside surveillance settings. CrowdHuman is characterized by extremely crowded scenes and heavy inter-person occlusion, and is therefore used to assess the model’s capability in dense pedestrian detection.

To further verify the cross-domain generalization ability of the proposed method, we also introduce the COCO person subset and the VisDrone2019 dataset. The COCO person dataset is extracted from the large-scale MS COCO benchmark and contains only images with person annotations. Owing to its diverse scene categories, complex backgrounds, and large pose variations, it provides an important benchmark for evaluating the model’s generalization beyond pedestrian-specific surveillance scenarios. In contrast, VisDrone2019 is collected from UAV platforms and represents aerial-view detection conditions. It contains a large number of small-scale targets, significant scale variation, and frequent occlusion, making it suitable for testing the model’s robustness in drone-based and cross-view surveillance tasks.

Overall, the custom-made Person dataset is used to evaluate the model in the complex scenarios targeted in this work, while the five public datasets are introduced to provide standardized benchmarks and to verify the robustness and generalization ability of RMP-YOLO across different viewpoints, scene distributions, and crowd densities.

### 4.2. Evaluation Indicators

Model evaluation is conducted along three axes: mAP (mean Average Precision), total parameters (Params), and GFLOPS. Recall *R* quantifies the percentage of missed samples, whereas Precision *P* indicates the percentage of false positive samples in the model output. The formulas are expressed as follows:

(11) $$P=\frac{TP}{TP+FP}$$

(12) $$R=\frac{TP}{TP+FN}$$

TP is defined as true positives, FP is defined as false positives, and FN represents the number of missed true samples. As shown in Table 2, AP refers to the average precision for a single class label, which is defined as follows:

(13) $$AP=\int_{0}^{1}\mathrm{Precision}\left(Recall\right)\;d\mathrm{Recall}$$

mAP is obtained by averaging the per-category AP values across all *K* classes; a larger AP signifies superior average precision and thus stronger overall detection capability. The formula of mAP is defined as follows:

(14) $$mAP=\frac{\sum_{k=1}^{K}AP_{k}}{K}$$

The computation of the intersection area between the ground truth box and the predicted bounding box is the primary component of IoU in the model performance evaluation phase. Selecting different IoU thresholds directly affects the determination of the prediction results. The prediction is deemed accurate if the IoU hits or surpasses the predetermined threshold. If the IoU falls below the threshold, the prediction is considered a failure. The model evaluation uses two mAP indicators: the precision metric at an IoU threshold of 0.5 and the comprehensive metric across IoU thresholds from 0.5 to 0.95. Weights, biases, and other changeable factors used in model training are collectively referred to as parameters. The parameter count measures both the model’s memory footprint and its architectural complexity. A reduction in the parameter scale directly reflects the model’s lightweight design.

The model’s lightweight nature is immediately reflected in the degree of parameter reduction. The number of floating-point operations used in forward propagation is measured in GFLOPS, a metric of computational complexity. This metric directly reflects the model’s processor resource consumption. Models with lower computational demands are generally more favorable in terms of model complexity and hardware resource consumption.

The experimental hardware and software environment used in this study is summarized in Table 3. All experiments were conducted on a Windows 11 platform equipped with an Intel^®^ Core™ i7-12700F CPU (Intel, Santa Clara, CA, USA) and an NVIDIA^®^ GeForce RTX™ 3060 GPU (Nvidia, Santa Clara, CA, USA). The software environment mainly included Python 3.9.21, PyTorch 2.1.1, and CUDA 11.8. In addition, the inference speed of the trained RMP-YOLO model was measured on the NVIDIA RTX 3060 under batch-size-1 inference with an input size of 640. After warm-up, the model achieved approximately 49 FPS, corresponding to an average inference time of about 20.27 ms per image.

To ensure a controlled comparison across datasets of different scales, all training settings were kept identical except for the total number of epochs. Specifically, 200 epochs were used for the Person dataset, while 150 epochs were used for all the other datasets considered in this study. This adjustment was made according to the dataset scale and computational cost. Except for the training duration, the remaining hyperparameters were kept unchanged. In addition, the optimizer configuration and learning-rate strategy followed the default YOLOv8 settings used in this work. The common training settings are summarized in Table 4.

In addition, the inference speed of the trained RMP-YOLO model was measured on an NVIDIA RTX 3060 under batch-size-1 inference with an input size of 640. After warm-up, the model achieved approximately 49 FPS, corresponding to an average inference time of about 20.27 ms per image.

### 4.3. Visualization Experiment

**Figure 4:**
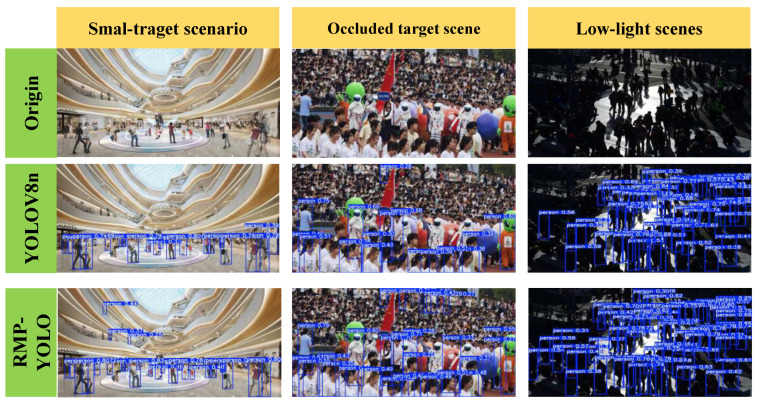
Comparison of pedestrian detection performance across three challenging scenarios: small-target scenario, occluded target scene, and low-light scene.

To underscore the exceptional performance of RMP-YOLO in challenging scenarios characterized by small objects, dense crowds, and low-light conditions, Figure 4 shows visualizations across these three settings. Furthermore, to elucidate the model’s robust anti-interference capabilities and resilience in dense crowd environments, we used Grad-CAM [43] to generate activation heatmaps for three representative cases. The regions in the heatmap that are redder indicate areas where the network pays more attention.

**Figure 5:**
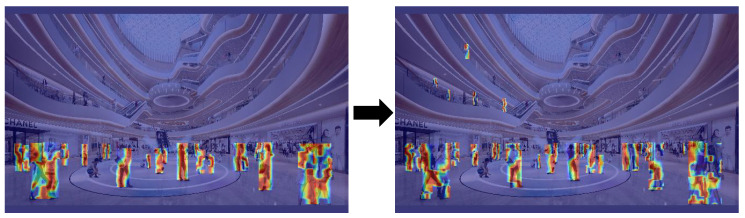
Heat map comparison between RMP-YOLO (right) and YOLOv8n (left) for small target pedestrian detection.

**Figure 6:**
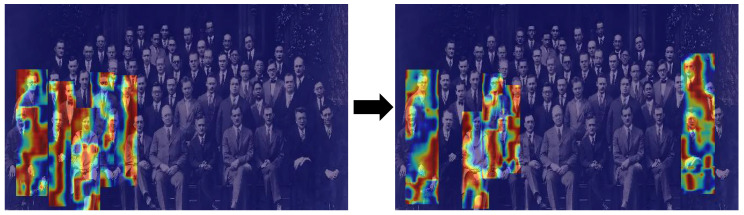
Heat map comparison between RMP-YOLO (right) and YOLOv8n (left) in dark, occluded target pedestrian detection.

Figure 5 and Figure 6 present the heatmaps produced by the model trained on the “Person” dataset. It is clear that RMP-YOLO shows a larger red area when detecting small and occluded targets. In Figure 5, although the pedestrians in the middle region remain unactivated due to severe feature loss caused by extreme low-light occlusion, RMP-YOLO successfully directs attention to the pedestrians on the left side. In contrast, the baseline YOLOv8n fails to focus on this area entirely. This indicates that, in terms of visualization, RMP-YOLO has improved the accuracy.

### 4.4. Dedicated Evaluation in Low-Light Conditions

To further investigate the performance of the proposed method under insufficient illumination, a dedicated low-light subset was constructed from the Person test set. Specifically, each test image was converted from RGB to YCbCr, and its mean luminance was computed from the Y channel. For the *i*-th image $I_{i}$ with spatial resolution $H_{i}\times W_{i}$, the mean Y-channel luminance is defined as

(15) $$\mu_{Y}^{\left(i\right)}=\frac{1}{H_{i}W_{i}}\sum_{x=1}^{H_{i}}\sum_{y=1}^{W_{i}}Y_{i}\left(x,y\right),$$

where $Y_{i}\left(x,y\right)$ denotes the luminance value of the pixel at position (x,y) in the Y channel. Candidate low-light samples were selected according to the following criteria:

(16) $$\mu_{Y}^{\left(i\right)}\leq 60.$$

Here, the threshold value 60 was defined on the 8-bit Y channel after inspecting the luminance statistics and representative scenes in the Person test set, to identify images that are globally illumination-deficient rather than those containing only locally dark regions. All candidate samples were then manually reviewed, and only those genuinely characterized by insufficient illumination, such as nighttime roads, dim indoor corridors, and strongly backlit pedestrian scenes, were retained in the final low-light subset. YOLOv8n and RMP-YOLO were evaluated on this subset using identical trained weights, inference settings, and evaluation criteria. The corresponding quantitative results are reported in Table 5.

As shown in Table 5, RMP-YOLO achieves better performance than YOLOv8n on the dedicated low-light subset in terms of mAP@0.5 and Recall. Specifically, mAP@0.5 increases from 83.8% to 84.7%, while Recall improves from 69.60% to 73.90%, indicating that the proposed method detects more pedestrian instances under insufficient illumination and reduces missed detections in challenging low-light scenes. Meanwhile, the increase in model complexity remains limited, with Params rising only from 3.15 M to 3.71 M and GFLOPs increasing from 4.10 to 6.29.

It is also observed that Precision decreases slightly from 88.10% to 87.20%, and mAP@0.5–0.95 changes marginally from 53.4% to 52.9%. This phenomenon is acceptable in difficult low-light detection tasks, where improving sensitivity to weak and ambiguous pedestrian cues often leads to higher Recall at the cost of a slight reduction in Precision. Therefore, the results suggest that the incorporation and fusion of the three components further improve the overall framework, enabling RMP-YOLO to maintain a stronger pedestrian detection capability under low-light conditions with only modest additional computational cost.

Combined with the qualitative results in Figure 4, these findings provide additional evidence that the proposed framework exhibits improved robustness in illumination-degraded pedestrian detection scenarios.

### 4.5. Ablation Experiment

To further evaluate the robustness and effectiveness of the proposed modules, ablation studies were conducted on the WiderPerson and Person datasets. Following the training settings described in Section 4.2, 150 epochs were used for WiderPerson, while 200 epochs were used for Person. The results are shown in Table 6 and Table 7.

By comparing the data in Table 6, RFAConv and MobileViTv3 outperform YOLOv8n, with improvements of 0.67% and 1.12% in mAP@0.5, respectively, and a slight increase of 0.28% and 0.66% in mAP@0.5–0.95, respectively. This demonstrates that both algorithms offer a noticeable improvement in average precision. According to the Recall metric, RMP-YOLO shows increases of 1.66% and 2.24% over YOLOv8n, reflecting its improved ability to predict true positives. For the Precision metric, RFAConv and MobileViTv3 both show a slight decrease compared to YOLOv8n, but the reduction is minimal. It should be noted that adding these three algorithms improves the model’s ability to identify different target characteristics, increasing the number of false positives and treating certain small or unclear targets, as well as occluded targets, as errors. However, for Recall, reducing false negatives improves Recall performance. Notably, the complexity overhead introduced by RFAConv is marginal. Compared with YOLOv8n, the parameter count increases only from 3.01 M to 3.11 M and the GFLOPs rise slightly from 4.10 to 4.22, while mAP@0.5 improves by 0.67% on WiderPerson and 0.47% on Person, demonstrating a favorable accuracy-efficiency trade-off.

Table 6 shows that PIoUv2 alone leads to a slight performance drop on the WiderPerson dataset. This suggests that the strict geometric penalty of PIoUv2 requires high-quality feature representations to be effective. Without the enhanced spatial and semantic features from RFAConv and MobileViTv3, the regression loss may over-penalize ambiguous samples. However, in the final RMP-YOLO configuration, PIoUv2 synergizes with the improved features to achieve the highest accuracy, validating the necessity of the complete architectural integration.

Furthermore, the RMP-YOLO model has demonstrated excellent performance on datasets such as Person, which involve single-labeled pedestrian targets. As shown in Table 7, all models achieve mAP@0.5 greater than 90%, indicating that high adaptability is achieved with 200 epochs. Additionally, RFAConv, MobileViTv3, and PIoUv2 show improvements in mAP@0.5 over YOLOv8n by 0.47%, 1.09%, and 0.24%, respectively, and improvements in mAP@0.5–0.95 by 0.69%, 1.42%, and 0.52%. These results represent a solid baseline for high accuracy. The Params and GFLOPs metrics for the Wider Person dataset are consistent with the results from this round of experiments. The Recall metrics show significant improvements across the board, with increases of 1.66%, 2.24%, and 1.18%. Comparing the results in Table 6 and Table 7 reveals that RFAConv and MobileViTv3 show identical improvements, while PIoUv2 shows a performance increase on the Person dataset, which uses a single-object label.

Regarding the RMP-YOLO model, as shown in Table 7, the mAP@0.5 and mAP@0.5–0.95 metrics achieve the highest values of 55.94% and 32.47%, respectively, compared to YOLOv8n and any individual fusion algorithm. Table 6 also shows that RMP-YOLO achieves the highest scores of 93.01% and 62.90% for these two metrics, underscoring the excellence of the RMP-YOLO algorithm and reflecting the effectiveness of module fusion in improving average detection accuracy. Moreover, leveraging MobileViTv3, the model maintains remarkable performance despite its compact parameter count. The Precision and Recall metrics in Table 6 and Table 7 show that although Precision decreases compared to the official model, it still achieves a high level of performance, demonstrating the effectiveness of module fusion in terms of Precision.

Further analysis of the ablation results reveals that the performance of RMP-YOLO is not merely a linear superposition of individual module gains but the result of significant synergistic effects grounded in theoretical complementarity. In deep learning model design, blindly stacking modules often leads to feature conflicts or diminishing returns. Our experiments confirm that RMP-YOLO achieves the best performance because the three introduced modules address distinct yet complementary theoretical bottlenecks in dense pedestrian detection. Specifically, RFAConv operates on the spatial dimension by dynamically adjusting the receptive field to capture fine-grained details of small targets. This provides a high-fidelity feature foundation that subsequent layers require. Building on this, MobileViTv3 operates on the semantic dimension by capturing global contextual dependencies to mitigate occlusion issues. It effectively utilizes the detailed features from RFAConv to infer object relationships that local convolutions cannot resolve. Finally, PIoUv2 operates on the geometric dimension by refining the localization precision through an optimized regression mechanism. It targets the coordinate uncertainty inherent in crowded overlaps, which feature enhancement alone cannot fix. This spatial-semantic-geometric complementarity enables the modules to function effectively across orthogonal dimensions without interfering with one another. Consequently, they serve as complementary components within a jointly optimized detection framework, thereby improving robustness and accuracy.

### 4.6. Controlled Experiment for Generalization Assessment

#### 4.6.1. Cross-Dataset Controlled Experiment

This study evaluates the generalization ability of the RMP-YOLO algorithm across multiple public datasets under the training settings described in Section 4.2, and the results are presented in Table 8.

On the Wider Person dataset, mAP@0.5 increased from 55.36% to 55.94%, and mAP@0.5–0.95 increased from 31.56% to 32.47%. Precision decreased slightly from 61.74% to 61.09%, while Recall increased from 49.86% to 52.37%, indicating improved target coverage at a small cost to precision.

On the Crowd Human dataset, mAP@0.5 increased from 74.21% to 77.09%, and mAP@0.5–0.95 increased from 45.89% to 49.22%. Precision improved from 83.13% to 84.38%, while Recall decreased from 63.72% to 60.01%. This suggests a precision–recall trade-off at the selected operating point. Despite the lower Recall, the gains in mAP indicate better overall detection quality on this dataset.

On the City Persons dataset, mAP@0.5 increased from 51.28% to 53.62%, and mAP@0.5–0.95 increased from 26.67% to 28.51%. Precision improved from 69.52% to 70.12%, and Recall improved from 43.08% to 45.27%, indicating fewer false positives and better true-positive coverage.

On the COCO person dataset, mAP@0.5 increased from 75.63% to 78.39%, and mAP@0.5–0.95 increased from 52.39% to 55.24%. Precision improved from 79.48% to 80.72%, and Recall improved from 66.02% to 69.25%, showing a clear overall improvement in detection performance.

On the VisDrone2019 dataset, mAP@0.5 increased from 40.40% to 42.00%, and mAP@0.5–0.95 increased from 16.98% to 17.67%. Precision improved from 55.30% to 56.01%, and Recall improved from 37.72% to 39.54%, demonstrating improved robustness in complex aerial-view scenarios.

Across multiple public datasets, RMP-YOLO consistently achieves higher mAP values than YOLOv8 and maintains competitive Precision and Recall, demonstrating good cross-dataset generalization.

#### 4.6.2. Test Set Validation Experiment

To further verify the generalization ability of the RMP-YOLO algorithm, the model trained on the Person dataset under the training settings described in Section 4.2 was used to perform inference on partial data from several public datasets. The results are shown in Table 9.

On the Wider Person test set, after inference on 1000 instances, mAP@0.5 increased from 60.9% to 62.7%, and mAP@0.5–0.95 increased from 28.0% to 29.8%. Precision improved from 81.6% to 83.1%, and Recall improved from 50.0% to 51.2%, indicating better overall detection performance.

On the Crowd Human validation set, after inference on 500 instances, mAP@0.5 increased from 33.5% to 36.3%, and mAP@0.5–0.95 increased from 15.5% to 16.8%. Precision improved from 59.2% to 59.5%, and Recall improved from 30.2% to 32.7%, indicating improved detection quality and stronger true-positive coverage.

On the City Persons validation set, after inference on 99,752 instances, mAP@0.5 increased from 52.0% to 53.6%, and mAP@0.5–0.95 increased from 23.8% to 24.7%. Precision improved from 73.0% to 74.3% and Recall from 42.4% to 44.3%, indicating better overall transfer performance on this dataset.

On the COCO person validation set, after inference on 2693 instances, mAP@0.5 changed from 37.5% to 36.9%, while mAP@0.5–0.95 changed from 16.0% to 16.1%. Precision changed from 61.4% to 59.3%, and Recall changed from 34.4% to 33.9%. These results indicate that the transfer advantage on this dataset is limited, although the high-IoU metric remains essentially stable.

On the VisDrone2019 validation set, after inference on 6412 instances, mAP@0.5 increased from 10.5% to 12.6%, and mAP@0.5–0.95 increased from 3.78% to 4.5%. Recall improved from 8.43% to 11.4%, while Precision decreased from 39.5% to 35.6%, indicating a precision–recall trade-off under this cross-dataset transfer setting.

Based on the overall validation results across these datasets, RMP-YOLO shows better transfer performance than YOLOv8 on most evaluated datasets, especially on Wider Person, Crowd Human, City Persons, and VisDrone2019. Although the improvement on COCO person is limited, the overall results still demonstrate favorable generalization of the proposed method.

#### 4.6.3. Comparative Evaluation Against Contemporary YOLO Variants

To further evaluate the competitiveness of the proposed method within the current YOLO series, comparative experiments were conducted on the public WiderPerson dataset using YOLOv8, YOLOv10, YOLOv11, and the proposed RMP-YOLO, and the corresponding results are reported in Table 10. The WiderPerson dataset was selected because it is a widely used public benchmark that contains diverse pedestrian scenes with large-scale variations, frequent occlusions, and complex backgrounds, thereby providing a more objective and convincing basis for performance evaluation in dense pedestrian detection tasks.

For a fair comparison, YOLOv8, YOLOv10, YOLOv11, and RMP-YOLO were all trained and evaluated on the same WiderPerson split under the unified training settings described in Section 4.2, namely 150 epochs, batch size 8, 8 workers, and an input image size of 640. All experiments were conducted on the same hardware platform reported in Table 3. It should also be noted that although YOLOv10 and YOLOv11 are more recent versions, newer YOLO variants do not necessarily achieve better accuracy on every task-specific benchmark. WiderPerson emphasizes dense pedestrian scenes with frequent occlusion, scale variation, and complex backgrounds. Under a unified training protocol without additional dataset-specific hyperparameter retuning, the architectural preferences of YOLOv10 and YOLOv11 may be less favorable than those of YOLOv8 on this particular benchmark.

As shown in Table 10, RMP-YOLO achieves the best overall detection performance among the compared models. Specifically, RMP-YOLO attains the highest mAP@0.5 of 55.94% and the highest mAP@0.5–0.95 of 32.47%, which are both superior to those of YOLOv8, YOLOv10, and YOLOv11. Compared with YOLOv8, the proposed method improves mAP@0.5 by 0.58 percentage points and mAP@0.5–0.95 by 0.91 percentage points. The gains become more evident when compared with YOLOv10 and YOLOv11, indicating that the proposed improvements are still effective when benchmarked against more recent YOLO variants.

From the perspective of precision–recall characteristics, RMP-YOLO also exhibits a more favorable performance balance. In pedestrian detection tasks, especially in dense, occluded crowd scenes, missed detections are usually more critical than a slight increase in false alarms; therefore, recall is of particular practical importance. In this regard, RMP-YOLO achieves the highest recall of 52.37%, exceeding YOLOv8, YOLOv10, and YOLOv11 by 2.51, 2.41, and 1.39 percentage points, respectively, indicating that the proposed model detects more pedestrian instances under challenging conditions. Meanwhile, the precision of RMP-YOLO reaches 61.09%, which is still higher than that of YOLOv10 and YOLOv11, and only slightly lower than YOLOv8. This suggests that the recall improvement is not obtained at the expense of a severe precision degradation, but rather through a better overall balance between false positives and missed detections.

In terms of model complexity, YOLOv10 and YOLOv11 reduce the number of parameters to $2.71\times 10^{6}$ and $2.59\times 10^{6}$, respectively, but their computational costs increase to 8.4 and 6.4 GFLOPs. In particular, YOLOv10 shows the most obvious increase in GFLOPs despite having fewer parameters. By comparison, RMP-YOLO contains $3.71\times 10^{6}$ parameters, which is moderately higher than the baseline models, but its computational cost is 6.29 GFLOPs, remaining lower than both YOLOv10 and YOLOv11. Therefore, although the proposed method introduces a limited increase in parameter scale, it still maintains a relatively favorable computational burden while delivering the best comprehensive detection performance.

Overall, the results above demonstrate that RMP-YOLO achieves a better trade-off among detection accuracy, recall, and computational complexity on the WiderPerson benchmark, further confirming its practical value for pedestrian detection in complex crowd scenes.

### 4.7. Model Training Capability Comparison

**Figure 7:**
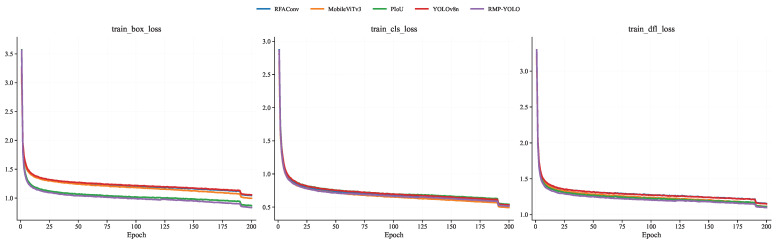
Comparison of training set loss function of different fusion modules under the Person data set.

**Figure 8:**
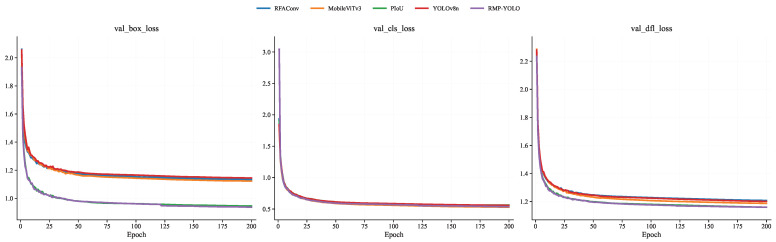
Comparison of loss functions of different fusion module verification sets under the Person data set.

**Figure 9:**
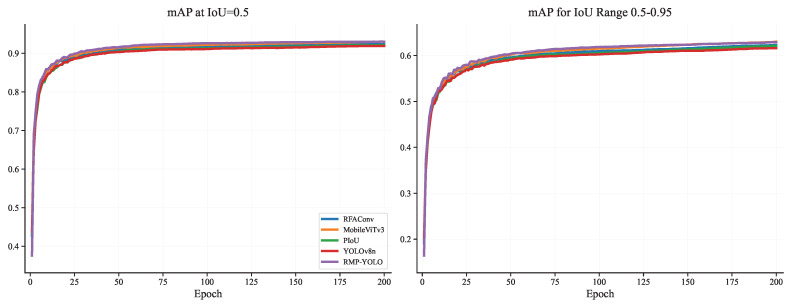
Comparison of mAP@0.5 and mAP@0.5–0.95 of different fusion modules under the Person data set.

**Figure 10:**
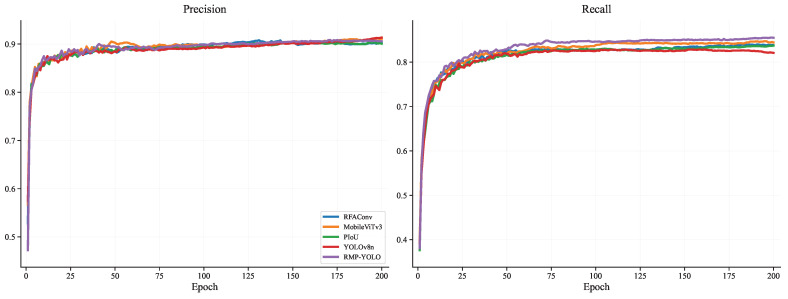
Comparison of accuracy and recall rates of different fusion modules under the Person data set.

To further analyze the training behavior of the proposed method, comparisons were conducted on the Person dataset in terms of training loss, validation loss, mAP@0.5, mAP@0.5–0.95, Precision, and Recall. The training-loss curves are shown in Figure 7, the validation-loss curves are shown in Figure 8, the mAP curves are shown in Figure 9, and the Precision/Recall curves are shown in Figure 10. “YOLOv8n” represents the baseline model used for comparison.

The training loss evaluates the model’s learning process and reflects its fit to the training data, while the validation loss primarily monitors generalization and potential overfitting. From the train_box_loss and train_dfl_loss curves in Figure 7, it is clear that all models exhibit good convergence trends during training, and convergence is essentially achieved around the 25th epoch. In addition, PIoUv2 and RMP-YOLO show relatively faster convergence. From the val_box_loss and val_dfl_loss curves in Figure 8, a similar trend can be observed, indicating that the proposed improvements also contribute to stable validation behavior during training.

In Figure 9, it can be seen that the average precision of the IoU at the 50% threshold for all models exceeds 90% by around the 40th epoch, and the performance of all fusion models is better than that of YOLOv8n. Notably, RMP-YOLO shows the best performance. After 150 epochs, the curves for MobileViTv3 and RMP-YOLO overlap closely, indicating that the fusion of the individual modules has led to a noticeable improvement. mAP@0.5–0.95, which measures the average precision across the 50–95% IoU threshold range, is a more stringent evaluation metric. It is evident that the fusion modules outperform YOLOv8, and RMP-YOLO stands out among them. Additionally, after the 150th epoch, MobileViTv3 surpasses RMP-YOLO, suggesting that the introduction of the multi-head attention mechanism is crucial for improving object recognition accuracy.

**Figure 11:**
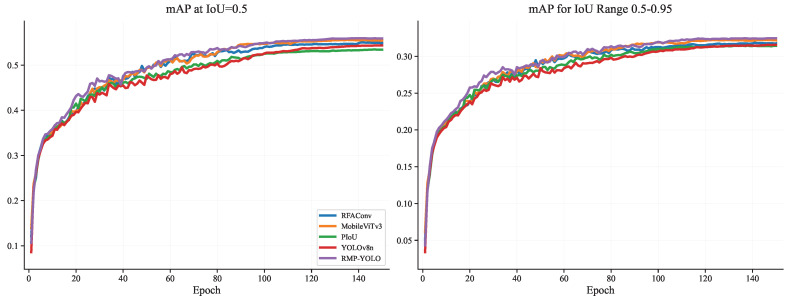
Comparison of mAP@0.5 and mAP@0.5-0.95 of different fusion modules under Wider Person.

**Figure 12:**
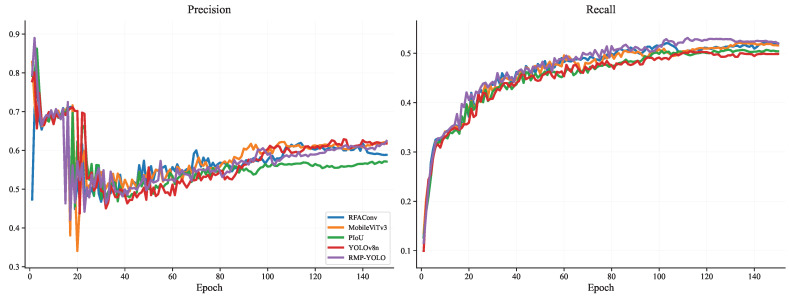
Comparison of Precision and Recall of different fusion modules under Wider Person.

Figure 10 shows that in terms of the Precision metric, the performance of the fusion modules is somewhat unstable. Before approximately the 75th epoch, MobileViTv3 outperforms the others. Between the 75th and 150th epochs, RFAConv performs better, while after the 175th epoch, YOLOv8n outperforms the others. Although YOLOv8n shows slightly higher Precision in the final training stages, this is an acceptable trade-off for the significant gains in Recall and mAP. Since RMP-YOLO is optimized for hard-to-detect samples, it becomes more sensitive, leading to a marginal increase in False Positives. In safety-critical applications like autonomous driving, minimizing False Negatives is prioritized over avoiding False Positives. Therefore, given the comprehensive improvements in mAP and Recall, RMP-YOLO demonstrates superior overall performance for the intended task. The comparison of mAP@0.5, mAP@0.5–0.95, Recall, and Precision across different fusion modules on the WiderPerson dataset is shown in Figure 11 and Figure 12.

Figure 11 shows that the overall recognition accuracy is lower, making the comparison of the fusion modules more pronounced. It can be seen that all fusion modules outperform YOLOv8n in terms of mAP@0.5 and mAP@0.5–0.95, with RFAConv and MobileViTv3 showing larger improvements compared to the original model. Furthermore, when combined, RMP-YOLO outperforms the individual modules, indicating that the modules are not significantly incompatible and that combining them achieves better overall accuracy.

About Precision, the results on the Wider Person dataset show significant complexity. From Figure 12, it can be seen that all models experience a sharp change in performance around the 20th epoch. In the first 100 epochs, the fused models outperform YOLOv8n; however, after the 100th epoch, YOLOv8n surpasses all fusion models, particularly PIoUv2, which performs poorly. This suggests that, for multi-class object recognition, the training efficiency of a loss function that emphasizes adaptive target locking is lower. However, an interesting result is that RMP-YOLO surpasses YOLOv8n after about 150 epochs, achieving a higher precision. MobileViTv3 also performs well in the overall Precision metrics. Overall, the MobileViTv3 module improves the accuracy of predicting positive samples.

In terms of Recall, the performance of the fusion modules remains quite similar to that observed on the Person dataset. Therefore, whether for single-label or multi-label recognition, fusion algorithms have improved the ability to correctly identify true samples.

Overall, the results above indicate that RMP-YOLO achieves a favorable balance between detection performance and model compactness in the same experimental setting. Compared with the YOLOv8n baseline, the proposed method improves overall detection effectiveness and robustness while maintaining a relatively compact architecture and moderate computational cost. These results demonstrate that the introduced modules enhance detection capability without imposing an excessive efficiency burden, thereby supporting the applicability of RMP-YOLO in resource-constrained pedestrian detection scenarios.

## 5. Conclusions

This study proposed RMP-YOLO, a lightweight, unified framework for dense pedestrian detection that addresses the coupled challenges of small-scale pedestrians, severe occlusion, and low-light conditions. Built upon YOLOv8, the proposed method integrates RFAConv for receptive-field-aware local feature extraction, MobileViTv3 for lightweight global–local context modeling, and PIoUv2 for geometry-aware bounding-box regression, thereby improving feature representation and localization quality in complex crowd scenes. Extensive experiments on the self-made Person dataset and multiple public benchmarks demonstrate that RMP-YOLO achieves strong detection performance while maintaining a compact model scale. In particular, it attains 55.94% mAP@0.5 and 32.47% mAP@0.5–0.95 on WiderPerson with only 3.71 M parameters and 6.29 GFLOPs, and also shows favorable cross-dataset generalization. In addition, under batch-size-1 inference on an RTX 3060 with an input size of 640, the trained model achieves 49.33 FPS, further supporting its practical deployment potential. These results indicate that the proposed framework provides an effective balance between detection accuracy, robustness, and computational efficiency, making it promising for practical applications such as surveillance systems, intelligent traffic monitoring, crowd safety analysis, and transportation hubs. Future work will focus on deployment-oriented runtime optimization on edge devices, extension to multi-camera collaborative environments, and further robustness enhancement through photorealistic simulated urban scenes, hybrid training, and domain adaptation, so as to better cover rare and difficult cases involving varying crowd density, occlusion, weather, viewpoints, and low-light conditions.

## Figures and Tables

**Table 1:** Dataset information.

Category	Training Set	Validation Set	Test Set	Label		
Person	14,728	1637	1819	person	–	–
Wider Person [38]	8000	4332	1000	pedestrians	riders	partially visible persons
City Persons [39]	2972	500	–	person	–	–
Crowd Human [40]	15,000	4370	–	head_box	visible_box	full_box
COCO person [41]	64,115	2693	–	person	–	–
VisDrone2019 [42]	6471	548	–	pedestrian	person	–

**Table 2:** Confusion matrix.

Actual Condition	Predicted Outcome	
	Positive	Negative
Positive	TP	FN
Negative	FP	TN

**Table 3:** Experimental Equipment.

Name	Version
Operating system	Windows 11
CPU	Intel^®^ Core™ i7-12700F @ 2.10 GHz
GPU	NVIDIA^®^ GeForce RTX™ 3060
Python	3.9.21
Pytorch	2.1.1
CUDA	11.8
VRAM	22 GB
RAM	16G

**Table 4:** Training settings summary.

Name	Version
Training parameter	Setting
Epoch (Person dataset)	200
Epoch (all other datasets)	150
Batch size	8
Image size	640
Workers	8

**Table 5:** Quantitative comparison on the low-light subset of the Person test set.

Model	Images	mAP@0.5/%	mAP@0.5–0.95/%	Params ($\times 10^{6}$)	P/%	R/%	GFLOPs
YOLOv8n	500	83.8	53.4	3.15	88.10	69.60	4.10
RMP-YOLO	500	84.7	52.9	3.71	87.20	73.90	6.29

**Table 6:** RMP-YOLO Ablation Experiments under the Wider Person Dataset, Note: ✓ indicates that the module is used, and – indicates that the module is not used, and the best results are shown in bold.

RFAConv	MobileViTv3	PIoUv2	mAP@0.5/%	mAP@0.5–0.95/%	Params ($\times 10^{6}$)	P/%	R/%	GFLOPs
–	–	–	54.36	31.56	3.01	61.74	49.86	4.10
✓	–	–	55.03	31.84	3.11	51.25	60.82	4.22
–	✓	–	55.48	32.22	3.71	61.78	51.90	6.29
–	–	✓	52.67	31.61	3.01	56.12	48.93	4.10
✓	✓	✓	**55.94**	**32.47**	**3.71**	**61.09**	**52.37**	**6.29**

**Table 7:** Ablation Experiments of RMP-YOLO in Person Dataset, Note: ✓ indicates that the module is used, and – indicates that the module is not used, and the best results are shown in bold.

RFAConv	MobileViTv3	PIoUv2	mAP@0.5/%	mAP@0.5–0.95/%	Params ($\times 10^{6}$)	P/%	R/%	GFLOPs
–	–	–	91.92	61.60	3.01	91.24	82.19	4.10
✓	–	–	92.39	62.29	3.11	90.33	83.85	4.22
–	✓	–	93.01	63.02	2.68	91.11	84.43	6.19
–	–	✓	92.16	62.12	3.01	90.08	83.73	4.10
✓	✓	✓	**93.01**	**62.90**	**3.71**	**90.61**	**85.53**	**6.29**

**Table 8:** Experimental results on different public datasets.

Dataset	Model	mAP@0.5/%	mAP@0.5–0.95/%	P/%	R/%
Wider Person	YOLOv8	55.36	31.56	61.74	49.86
	RMP-YOLO	55.94	32.47	61.09	52.37
Crowd Human	YOLOv8	74.21	45.89	83.13	63.72
	RMP-YOLO	77.09	49.22	84.38	60.01
City Persons	YOLOv8	51.28	26.67	69.52	43.08
	RMP-YOLO	53.62	28.51	70.12	45.27
COCO person	YOLOv8	75.63	52.39	79.48	66.02
	RMP-YOLO	78.39	55.24	80.72	69.25
VisDrone2019	YOLOv8	40.40	16.98	55.30	37.72
	RMP-YOLO	42.00	17.67	56.01	39.54

**Table 9:** Experimental results on validation sets on different datasets.

Dataset	Model	Instance	mAP@0.5/%	mAP@0.5–0.95/%	P/%	R/%
Wider Person	YOLOv8	1000	60.9	28.0	81.6	50.0
	RMP-YOLO		62.7	29.8	83.1	51.2
Crowd Human	YOLOv8	500	33.5	15.5	59.2	30.2
	RMP-YOLO		36.3	16.8	59.5	32.7
City Persons	YOLOv8	99,752	52.0	23.8	73.0	42.4
	RMP-YOLO		53.6	24.7	74.3	44.3
COCO person	YOLOv8	2693	37.5	16.0	61.4	34.4
	RMP-YOLO		36.9	16.1	59.3	33.9
VisDrone2019	YOLOv8	6412	10.5	3.78	39.5	8.43
	RMP-YOLO		12.6	4.5	35.6	11.4

**Table 10:** Comparative results of different YOLO variants on the WiderPerson dataset.

Dataset	Model	mAP@0.5%	mAP@0.5–0.95%	P%	R%	Params ($\times 10^{6}$)	GFLOPs
Wider Person	YOLOv8	55.36	31.56	61.74	49.86	3.01	4.10
	YOLOv10	52.00	31.40	57.30	49.96	2.71	8.4
	YOLOv11	53.67	31.16	60.40	50.98	2.59	6.4
	RMP-YOLO	55.94	32.47	61.09	52.37	3.71	6.29

## Data Availability

Publicly available datasets were analyzed in this study. These data can be found at the following locations: WiderPerson (http://www.cbsr.ia.ac.cn/users/sfzhang/WiderPerson/ (accessed on 19 April 2026)); CityPersons (https://github.com/cvgroup-njust/CityPersons (accessed on 19 April 2026)); CrowdHuman (http://www.crowdhuman.org/ (accessed on 19 April 2026)); COCO2017-Person (https://cocodataset.org/ (accessed on 19 April 2026)); and VisDrone2019 (https://github.com/VisDrone/VisDrone-Dataset (accessed on 19 April 2026)). The self-made dataset presented in this study is available on request from the corresponding author.
